# Supplementary material for: Spatiotemporal expression of regulatory kinases directs the transition from mitosis to cellular morphogenesis in Drosophila
Source: Nat Commun. 2022 Feb 9;13:772. doi: 10.1038/s41467-022-28322-8 (PMC8828718; doi:10.1038/s41467-022-28322-8)
Supplement: Supplementary file 1 — Supplementary information [file 41467_2022_28322_MOESM1_ESM.pdf]

Supplementary Information

Spatiotemporal expression of regulatory kinases directs the transition from mitosis to cellular morphogenesis in *Drosophila*

Shuo Yang[1], Jennifer McAdow[1], Yingqiu Du[1], Jennifer Trigg[2], Paul H. Taghert[2], and Aaron N. Johnson[1,3]

[1]Department of Developmental Biology
[2]Department of Neuroscience
Washington University School of Medicine
St. Louis, MO 63110

[3]Author for correspondence: anjohnson@wustl.edu
Running Title: Bsd activates Polo during myogenesis

Supplementary Figures

Supplementary Figure 1, related to Figure 1. (A) *in silico* expression analysis of *Drosophila* and zebrafish protein kinases. Embryonic expression patterns for all kinases were queried in Flybase and Zfin, and those with reported expression patterns were graphed. Expression patterns were subdivided by embryonic stage (Stages 0-5 in *Drosophila* occur prior to gastrulation), and classified as Tissue/Group Enriched, (meaning the kinase was expressed in a single tissue or a group of tissues but not all tissues), Ubiquitous, or Low Expression (in which the expression pattern was too faint to categorize). No Data indicates the expression pattern is known for only a subset of embryonic stages. See Table S1 for individual expression patterns. (B) *bsd* deficiency mapping. Live Stage 16 embryos that expressed Myosin Heavy Chain (MHC)-GFP. *bsd*^{1}/*Df(2R)BSC199* and *bsd*^{1}/*Df(2R)BSC699* embryos phenocopied *bsd*^{1} embryos. A control (*w*^{1118}) embryo is shown for comparison. (C) Phylogenetic analysis of human, mouse, and fly Vrk proteins. The homology tree was generated using observed divergency as the distance method. The percent homology among species is shown. (D) Protein alignment of the Vrk proteins from (C); black shading shows identical residues in all species, pink shading shows residues that are the same in most species, blue shading shows residues that are the same in at least 50% of species. The position of the *bsd*^{1} Q545* mutation is given (red box). Residues in the previously defined Kinase Domain (orange line) and ATP binding pocket (violet) for human Vrk1 are shown. Note that the Bsd kinase domain is split by a unique linker region (green line), and the C-termini of the Vrk proteins show low sequence homology. (E) Two-sided Fisher's exact test of the myogenic phenotypes shown in Fig. 1B. Significance indicates pairwise comparison between two genotypes. (*wt*, n=60; *bsd*^{1}, n=54; *bsd*^{1}*,61F090*, n=60; *bsd*^{1}*,61F090 out cross*, n=60). (F) Stage 12 *5053>GFP* and *slou>GFP* embryos labeled for GFP. Control (*5053>GFP*, n=7; *slou>GFP*, n=53) and *bsd*^{1} (*5053>GFP*, n=6; *slou>GFP*, n=35) embryos showed an equivalent number of GFP+ founder cells. Significance was determined by two-sided unpaired student's t test. Error bars represent SEM.

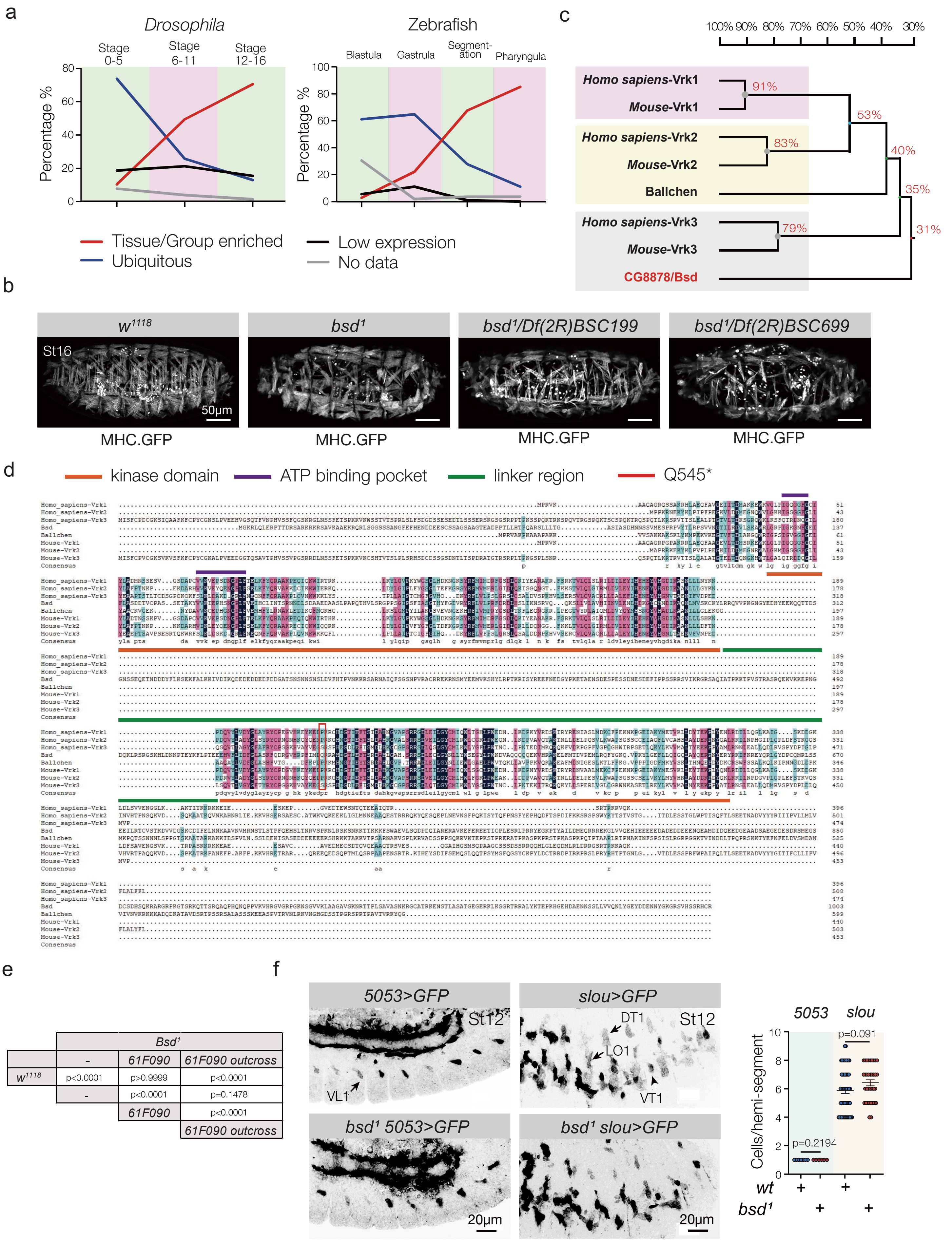
a
Drosophila
Stage 0-5
Stage 6-11
Stage 12-16
Percentage %
80
60
40
20
0
Zebrafish
Blastula
Gastrula
Segment-ation
Pharyngula
Percentage %
100
80
60
40
20
0
Tissue/Group enriched
Ubiquitous
Low expression
No data
c
100% 90% 80% 70% 60% 50% 40% 30%
Homo sapiens-Vrk1
Mouse-Vrk1
91%
53%
Homo sapiens-Vrk2
Mouse-Vrk2
83%
40%
Ballchen
35%
Homo sapiens-Vrk3
Mouse-Vrk3
79%
31%
CG8878/Bsd
b
w1118
St16
50µm
MHC.GFP
bsd1
MHC.GFP
bsd1/Df(2R)BSC199
MHC.GFP
bsd1/Df(2R)BSC699
MHC.GFP
d
kinase domain
ATP binding pocket
linker region
Q545*
Homo_sapiens-Vrk1
Homo_sapiens-Vrk2
Homo_sapiens-Vrk3
Bsd
Ballchen
Mouse-Vrk1
Mouse-Vrk2
Mouse-Vrk3
Consensus
e
Bsd1
-
61F090
61F090 outcross
w1118
p<0.0001
p>0.9999
p<0.0001
-
p<0.0001
p=0.1478
61F090
p<0.0001
61F090 outcross
f
5053>GFP
St12
VL1
slou>GFP
DT1
LO1
VT1
St12
bsd1 5053>GFP
20µm
bsd1 slou>GFP
20µm
5053
slou
p=0.091
p=0.2194
Cells/hemi-segment
10
8
6
4
2
0
wt
bsd1
+
+
+
+

Supplementary Figure 2, related to Figure 2. (A-C) Validation of a Bsd antibody. (A) Western blots of control (*w*[1118]), *Df(2R)BSC199,* and *rp298>Bsd* embryo lysates using the Bsd antibody. UAS/Gal4 expressed Bsd was detectable, but we could not detect endogenous Bsd by Western blot. (B) Western blot of S2 cell lysates transfected with control or Bsd.Myc expressing plasmids. The Bsd and Myc antibodies recognized proteins of the same molecular weight. (C) Stage 12 embryos immunolabeled with the Bsd antibody (green) and Mef2 (violet). Signal from the Bsd antibody was not observed in *Df(2R)BSC199* body wall muscle precursors. The embryos shown were prepared in parallel. (D) Complete embryonic expression of Bsd. Embryos were immunolabeled as in (C). Multiple confocal projections are shown for each embryo to compare expression in the ectoderm and the mesoderm. Bsd was ubiquitously expressed prior to gastrulation (Stage 5), and Bsd expression remained high in the ectoderm after gastrulation. Bsd expression in the mesoderm increased from Stage 11 to Stage 12, and then decreased. (E) Relative Bsd expression in the mesoderm, normalized to Stage 11 (St11, n=215; St12, n=421; St14, n=286; St16, n=292). (F) Embryos immunolabeled with the Bsd antibody (green) and membrane localized *Mef2>CD8.RFP* (violet). Bsd localization was enriched in the nucleus of Mef2+ cells through Stage 14. Significance was determined by one-way ANOVA. Error bars represent SEM.

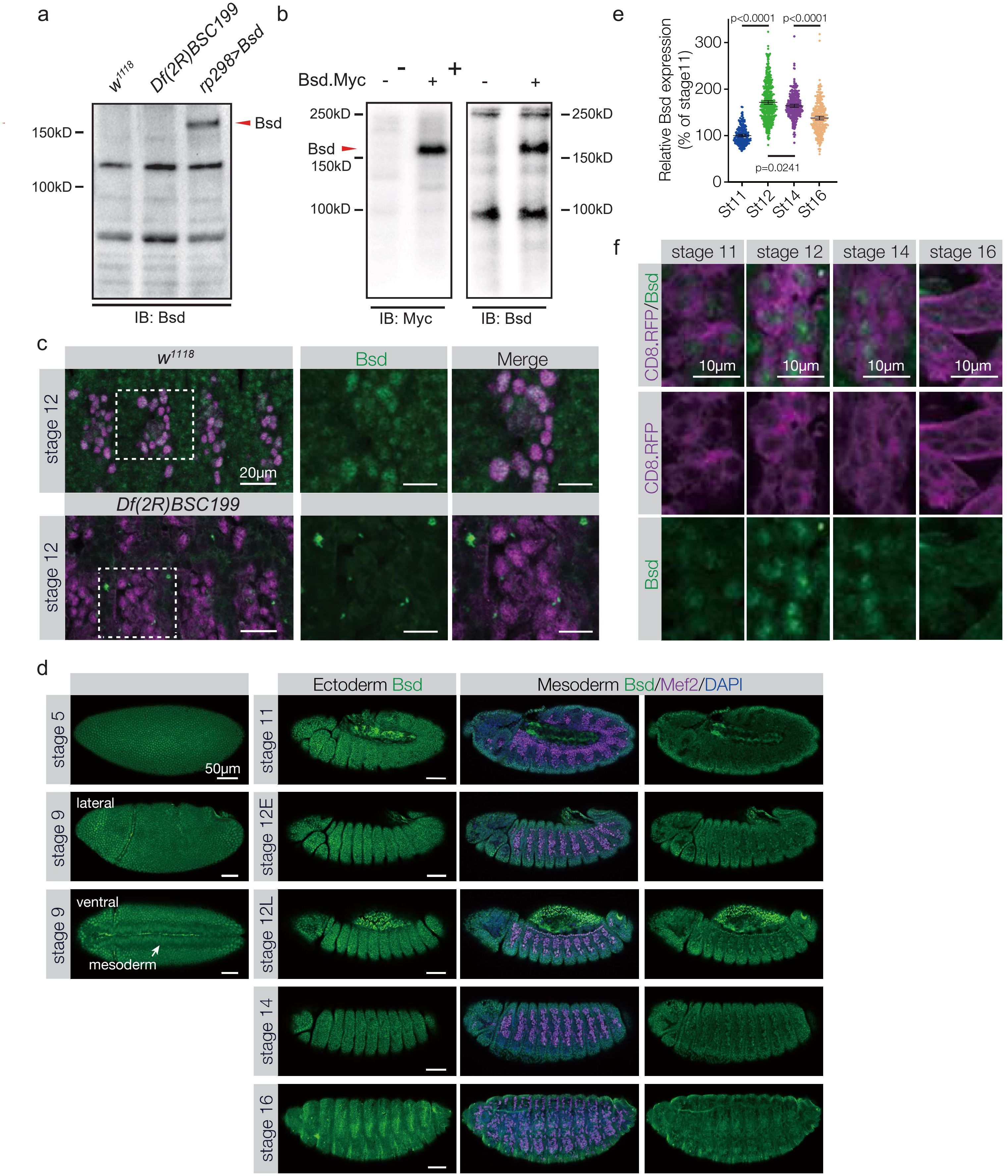

a
w1118
Df(2R)BSC199
rp298>Bsd
Bsd
150kD
100kD
IB: Bsd
b
Bsd.Myc
- +
250kD
Bsd
150kD
100kD
IB: Myc
IB: Bsd
c
w1118
Bsd
Merge
stage 12
20µm
Df(2R)BSC199
d
stage 5
50µm
lateral
stage 9
ventral
mesoderm
Ectoderm Bsd
Mesoderm Bsd/Mef2/DAPI
stage 11
stage 12E
stage 12L
stage 14
stage 16
e
Relative Bsd expression (% of stage11)
p<0.0001
p=0.0241
St11
St12
St14
St16
f
stage 11
stage 12
stage 14
stage 16
CD8.RFP/Bsd
CD8.RFP
10µm

Supplementary Figure 3, related to Figure 3. (A) Coomassie stained control gel for the GST-based precipitation experiment described in (Fig. 3A). Bsd-bound GST beads (lane 5; red shading) recovered a large number of unique bands compared to control GST beads (lane 3; blue shading). (B) Immunoprecipitation of S2 cell lysates transfected with Bsd and candidate proteins identified by MS. Bsd interacted with all five proteins, but Polo showed the strongest interaction. (C) Myogenic phenotypes in *polo* mutants. Stage 16 embryos labeled with Tropomyosin. DT1, LO1, and VL1 muscles are pseudocolored orange, green, and violet. The *polo* alleles showed stronger phenotypes in trans to *Df(3L)BSC447*, as expected for hypomorphic alleles. Heat maps and Fisher's exact test of the myogenic phenotypes are as described in Figs. 1B and S1E. (D) Histogram of *polo*^{1} VL1 phenotypes (n=54). Significance was determined by Fisher's exact test. (E) Stage 10 control embryos and *polo*^{1} embryos labeled for Mef2. (F) Statistical analysis of embryos shown in (E) (For each group, n=24). Each data point represents the number of Mef2+ cells in a single hemisegment; *polo*^{1} embryos had 14.2% fewer Mef2+ cells than controls. The vertical lines in the boxplots indicate the median, the boxes indicate the first and third quartiles, and the whiskers indicate minima and maxima. (G) Stage 12 *5053>GFP* embryos and *slou>GFP* embryos labeled for GFP. Control (*5053>GFP*, n=7; *slou>GFP*, n=50) and *polo*^{1} (*5053>GFP*, n=6; *slou>GFP*, n=39) embryos showed an equivalent number of GFP+ cells. (H) Statistical analysis of embryos shown in (G). Significance was determined by two-sided unpaired student's t test. (I) Two-sided Fisher's exact test of the myogenic phenotypes shown in Fig. 3F (*bsd*^{1}, n=54; *polo*^{1}, n=96; *bsd*^{1};*polo*^{1}, n=60). Error bars represent SEM.

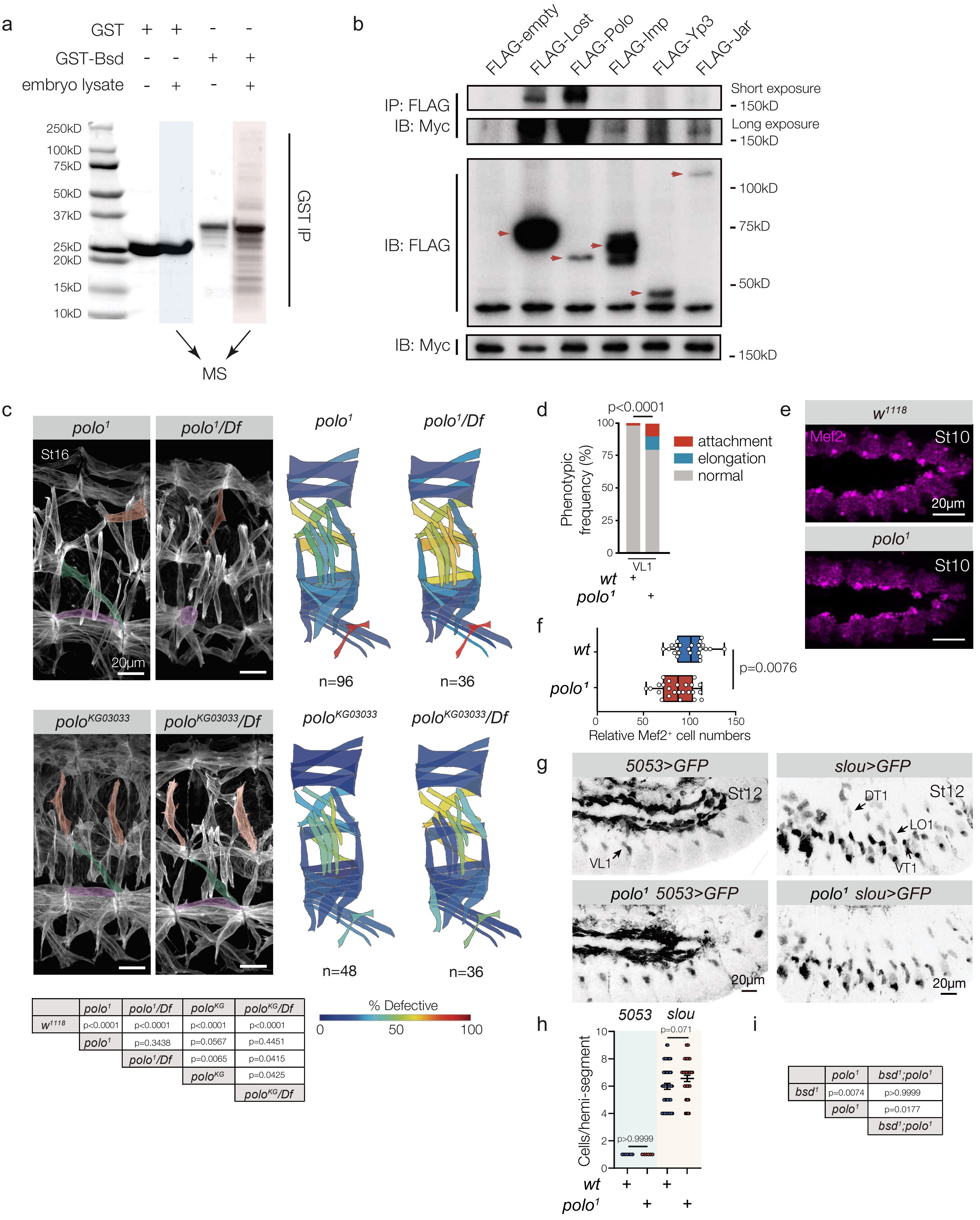

	$polo^1$	$polo^1/Df$	$polo^{KG}$	$polo^{KG}/Df$
w^{1118}	p<0.0001	p<0.0001	p<0.0001	p<0.0001
	$polo^1$	p=0.3438	p=0.0567	p=0.4451
		$polo^1/Df$	p=0.0065	p=0.0415
			$polo^{KG}$	p=0.0425
				$polo^{KG}/Df$

	$polo^1$	$bsd^1;polo^1$
bsd^1	p=0.0074	p>0.9999
	$polo^1$	p=0.0177
		$bsd^1;polo^1$

Supplementary Figure 4, related to Figure 4. (A) Lysates from S2 cells transfected with a control plasmid or a plasmid expressing Bsd.Myc were immunoblotted with anti-phosphothreonine (pThr). Lysates showed equivalent levels of pThr. (B) Lysates from S2 cells transfected with Polo and a control plasmid or Bsd.I129A were immunoblotted with pThr. The amount of phosphorylated Polo was not significantly different between cells transfected with Bsd.I129A and controls. (C) Lysates from S2 cells transfected with Polo and a control plasmid or wild-type Bsd were immunoblotted with anti-PLK1-phospho-T210 ($Polo^{pT182}$). There was a significant increase in the amount of phosphorylated Polo in cells transfected with Bsd compared to controls. (D) S2 cells transfected with Bsd.Myc and Polo, immunolabeled for pThr210-PLK1 ($Polo^{pT182}$ green) and Myc (red). Bsd transfected cells (arrows, n=45) showed significantly more $Polo^{pT182}$ than controls (n=76). (E) Purified GST-Bsd and GST-Polo were used for cell free *in vitro kinase* assays. $Polo^{pT182}$ was detected by immunoblotting with anti-pThr210-PLK1 antibody. Bsd directly phosphorylated Polo. (F) Early and late Stage 12 embryos immunolabeled for activated $Polo^{pT182}$ (green) and Mef2 (violet). $Polo^{pT182}$ increased during Stage 12 in control (*bsd*^{1} heterozygous) but not *bsd*^{1} embryos. Control and *bsd*^{1} embryos were labeled in the same preparation. (G) Stage 12 embryos with endogenous GFP-tagged Polo (*Polo*^{GFP}) that expressed *rP298.nLacZ* were immunolabeled for GFP (green) and lacZ (red). Fluorescent intensity and cellular localization of total Polo protein was comparable between control and *bsd*^{1} embryos. Graph plots Polo and nlacZ fluorescent intensity across myoblasts (n≥32 cells from 6 or more embryos per genotype). The median and
interquartile ranges are shown (dotted horizontal lines). Significance was determined by two-sided unpaired student’s t test (B-E). Error bars represent SEM.

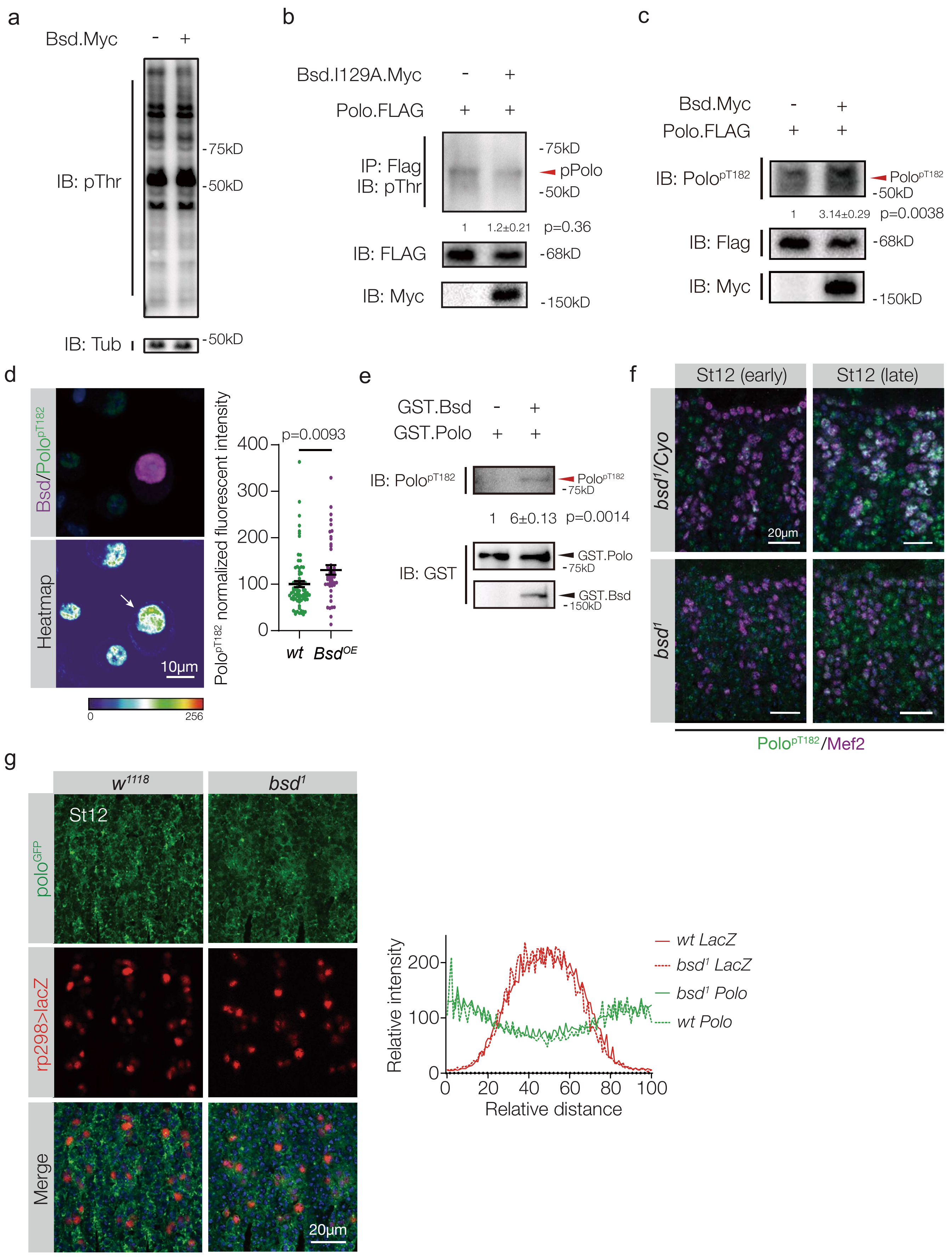

a
Bsd.Myc - +
IB: pThr
-75kD
-50kD
IB: Tub
-50kD
b
Bsd.I129A.Myc - +
Polo.FLAG + +
IP: Flag
IB: pThr
-75kD
pPolo
-50kD
1 1.2±0.21 p=0.36
IB: FLAG -68kD
IB: Myc -150kD
c
Bsd.Myc - +
Polo.FLAG + +
IB: PolopT182
PolopT182
-50kD
1 3.14±0.29 p=0.0038
IB: Flag -68kD
IB: Myc -150kD
d
Bsd/PolopT182
Heatmap
10µm
0 256
PolopT182 normalized fluorescent intensity
p=0.0093
400
300
200
100
0
wt BsdOE
e
GST.Bsd - +
GST.Polo + +
IB: PolopT182
PolopT182
-75kD
1 6±0.13 p=0.0014
IB: GST
GST.Polo
-75kD
GST.Bsd
-150kD
f
St12 (early)
St12 (late)
bsd1/Cyo
bsd1
20µm
PolopT182/Mef2
g
w1118
bsd1
St12
poloGFP
rp298>lacZ
Merge
20µm
Relative intensity
200
100
0
0 20 40 60 80 100
Relative distance
wt LacZ
bsd1 LacZ
bsd1 Polo
wt Polo

Supplementary Figure 5, related to Figure 5. (A) Stage 12 *rP298.nLacZ* embryo immunolabeled for phosphorylated Histone 3 (pH3, green) and lacZ (violet). lacZ expressing nascent myotubes were not pH3 positive. (B) Stage 12 embryos labeled for *aurA* (left, green), *aurB* (right, green) and Mef2 (violet). *aurA* and *aurB* expression was excluded from the somatic mesoderm in Stage 12 embryos. (C) Stage 16 embryos labeled with Tropomyosin. DT1, LO1, and VL1 muscles are pseudocolored orange, green, and violet. Muscle patterning was unaffected in $aurA^{1}$ and $aurB^{1689}$*/Df(3L)BSC447* embryos. (D) Quantification of muscle phenotypes, as described in Fig. 1B. (E) Stage 11 embryos immunolabeled for pH3 (green) and Mef2 (violet). *Mef2>aurB* embryos had significantly more pH3/Mef2 double positive cells than control embryos. n≥7 embryos per genotype. (F) Quantification of pH3/Mef2 double positive cells in (E). Each data point represents a single hemisegment (*wt*, n=80; *Mef2>aurB*, n=56). The median and interquartile ranges are shown (dotted horizontal lines). (G) Quantification of pH3/Mef2 double positive cells in (E), grouped by hemisegment (A2-A9) (n=10 embryos for wild type; n=7 embryos for *Mef2>aurB*). Error bars represent SEM. (H) Stage 16 embryos immunolabeled for Mef2 (green) and Tropomyosin (violet). (I) Myotubes in *Mef2>aurB* embryos had significantly more myonuclei than control embryos. n=6 embryos per genotype. The median and interquartile ranges are shown (dotted horizontal lines). Significance was determined by two-sided Fisher's exact test (D), two-sided unpaired student's t test (E,H), and two-way ANOVA (F). Error bars represent SEM.

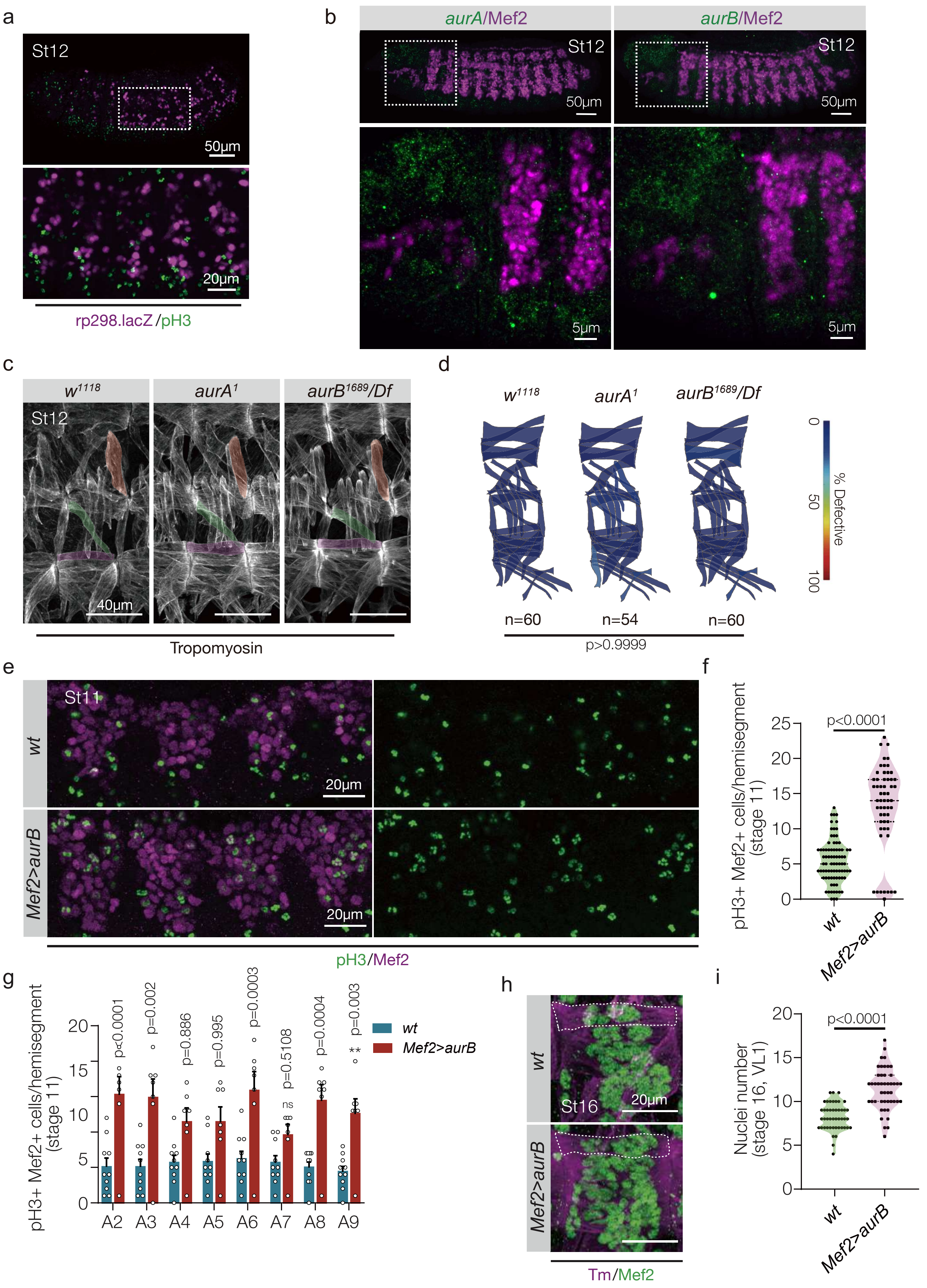

a
St12
50µm
20µm
rp298.lacZ/pH3
b
aurA/Mef2
aurB/Mef2
St12
50µm
5µm
c
w1118
aurA1
aurB1689/Df
St12
40µm
Tropomyosin
d
n=60
n=54
n=60
p>0.9999
% Defective
0
50
100
e
St11
wt
Mef2>aurB
20µm
pH3/Mef2
f
pH3+ Mef2+ cells/hemisegment (stage 11)
p<0.0001
wt
Mef2>aurB
g
p<0.0001
p=0.002
p=0.886
p=0.995
p=0.0003
p=0.5108
ns
p=0.0004
p=0.003
**
wt
Mef2>aurB
A2
A3
A4
A5
A6
A7
A8
A9
h
St16
20µm
Tm/Mef2
i
Nuclei number (stage 16, VL1)
p<0.0001

Supplementary Figure 6, related to Figure 6. (A) Stage 16 embryos labeled for *slou>GFP* (green) and Tropomyosin (violet). DT1, LO1, and VT1 muscle patterning was disrupted in *tum*^{DH15}, *polo*^{1}, and *tum*^{DH15} *polo*^{1} embryos. (B) Histogram of DT1, LO1, and VT1 phenotypes. The frequency of muscle phenotypes was not significantly different between *tum*^{DH15} embryos (n=54) and *tum*^{DH15} *polo*^{1} double mutant (n=42) embryos. For *polo*^{1} embryos, n=60. (C) Stage 16 embryos labeled with Tropomyosin. DT1, LO1, and VL1 muscles are pseudocolored orange, green, and violet. The muscle pattern was disorganized in *pav*^{B200} embryos, but the frequency of muscle phenotypes was not significantly different between *pav*^{B200} embryos and *tum*^{DH15} *pav*^{B200} embryos. n=36 hemisegments per genotype. Quantification of muscle phenotypes is as described in Fig. 1B. (D) LO1 myotubes from (Fig. 6C) showing F-actin expression by heat map. Scale bar represents the detection range. F-actin levels were equivalent between control and *bsd*^{1} myotubes. (E) Mononucleate LO1 myotubes from Stage 12 *slou>GFP* embryos labeled for γ-tubulin (green), GFP (violet) and DAPI (blue). Mononucleate, polarized myotubes have just initiated myotube guidance (see Movie 1). Control (*bsd*^{1}*/Cyo*) myotubes showed only cortical γ-tubulin foci. *bsd*^{1} myotubes had an equivalent number of cortical γ-tubulin foci compared to controls. (F) Quantification of γ-tubulin foci in mononucleate Stage 12 myotubes. Embryos used for (E,F) were derived from the same preparation. Significance was determined by two-sided Fisher's exact test (B,C), and one-way ANOVA (F). Error bars represent SEM.

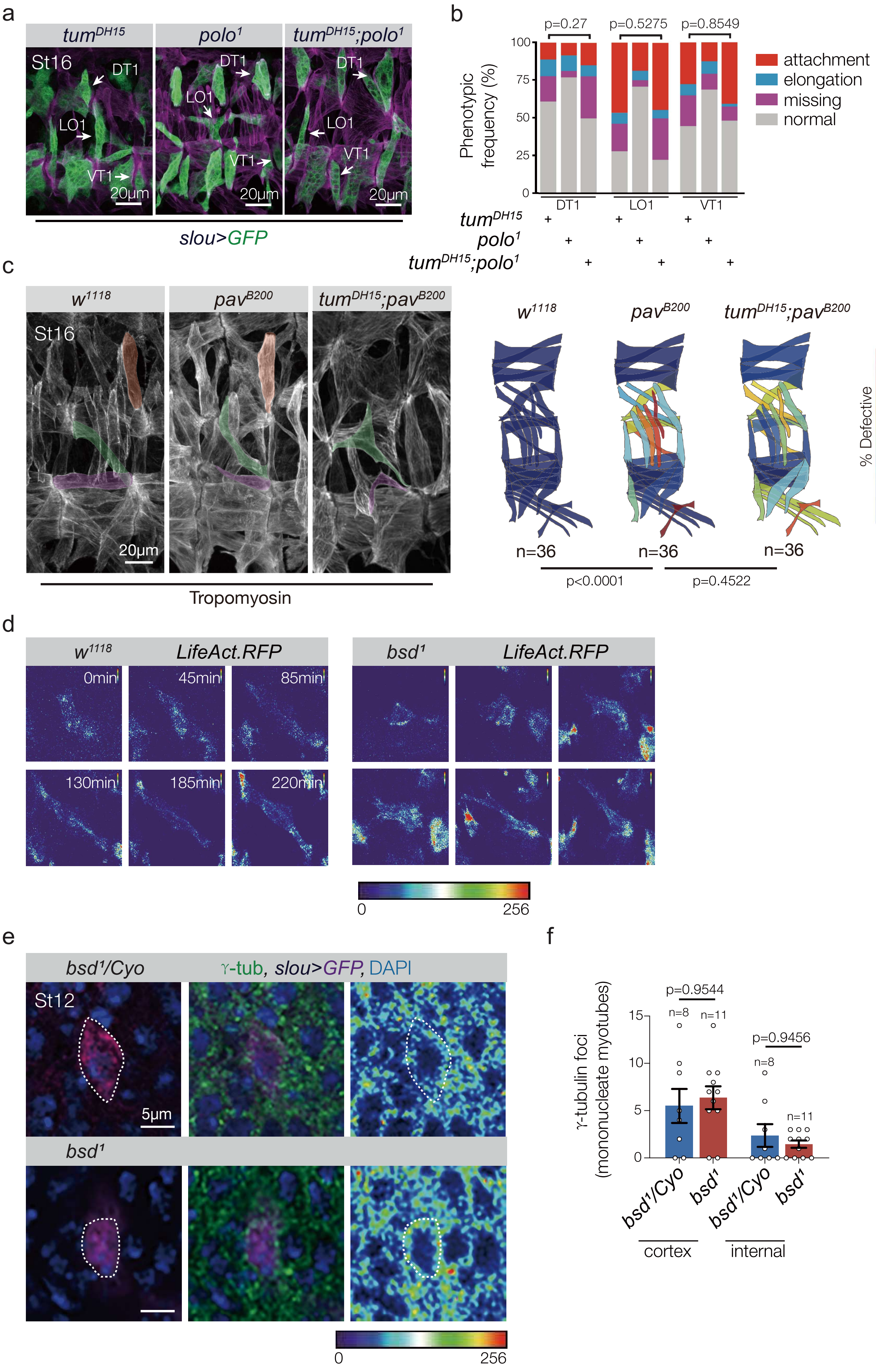
a
tumDH15
polo1
tumDH15;polo1
St16
DT1
LO1
VT1
20µm
slou>GFP
b
p=0.27
p=0.5275
p=0.8549
Phenotypic frequency (%)
100
75
50
25
0
DT1
LO1
VT1
attachment
elongation
missing
normal
tumDH15
polo1
tumDH15;polo1
c
w1118
pavB200
tumDH15;pavB200
St16
20µm
Tropomyosin
n=36
n=36
n=36
p<0.0001
p=0.4522
% Defective
0
50
100
d
w1118
LifeAct.RFP
bsd1
LifeAct.RFP
0min
45min
85min
130min
185min
220min
0
256
e
bsd1/Cyo
γ-tub, slou>GFP, DAPI
St12
5µm
bsd1
0
256
f
γ-tubulin foci (mononucleate myotubes)
p=0.9544
p=0.9456
n=8
n=11
n=8
n=11
15
10
5
0
bsd1/Cyo
bsd1
bsd1/Cyo
bsd1
cortex
internal

Supplementary Figure 7, related to Figure 7. (A) C2C12 cells treated with control (scrambled) siRNA or a cocktail of two siRNAs against murine Vrk1, Vrk2, or Vrk3. Cells were fixed after 7 days in differentiation media and labeled for α-actinin (green) to detect differentiated myotubes and DAPI (blue). Vrk3 knockdown myotubes were shorter than controls and often rounded. Vrk1 and Vrk2 knockdown myotubes were indistinguishable from control cells. (B) Quantitative real time PCR of siRNA treated C2C12 cells. Fold change is relative to control treated cells (red line) (VRK1 siRNA, n=2; VRK2 siRNA, n=2; VRK3 siRNA, n=3). (C) Myoblast fusion index shows reduced fusion in Vrk3 siRNA treated C2C12 cells. For each group, n=10. (D) QQ plot of myotube length distribution, related to Figure 7C. Each data point represents the actual length distribution value (x) and the predicted length value for a normal distribution (y). The lengths of the con siRNA (n=13) and DMSO (n=12) treated myotubes fit the normal distribution; the lengths of the Vrk3 siRNA (n=12) and Volasertib (n=10) treated myotubes did not fit the normal distribution. (E) Model of Bsd and the Polo/Tum/Pav cytoskeletal regulatory module. Bsd reorganizes the microtubule cytoskeleton to direct myotube guidance, and likely acts through Tum and Pav. Significance was determined by two-sided unpaired student’s t-test (C) and D'Agostino & Pearson test (D). Error bars represent SEM.

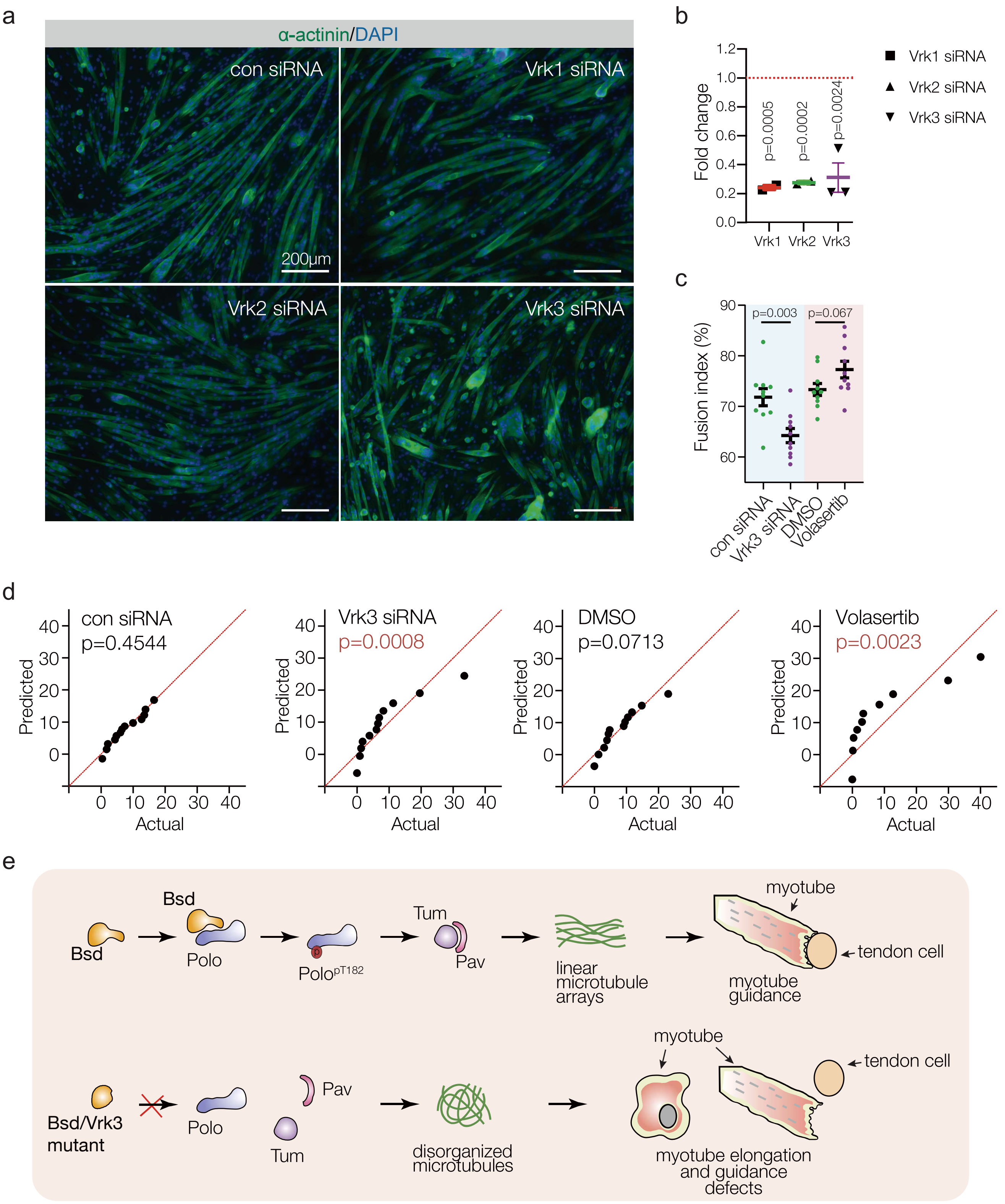

a
α-actinin/DAPI
con siRNA
Vrk1 siRNA
Vrk2 siRNA
Vrk3 siRNA
200μm
b
Fold change
p=0.0005
p=0.0002
p=0.0024
Vrk1 Vrk2 Vrk3
Vrk1 siRNA
Vrk2 siRNA
Vrk3 siRNA
c
Fusion index (%)
p=0.003
p=0.067
con siRNA
Vrk3 siRNA
DMSO
Volasertib
d
con siRNA
p=0.4544
Vrk3 siRNA
p=0.0008
DMSO
p=0.0713
Volasertib
p=0.0023
Predicted
Actual
e
Bsd
Polo
Polo^{pT182}
Tum
Pav
linear microtubule arrays
myotube
tendon cell
myotube guidance
Bsd/Vrk3 mutant
disorganized microtubules
myotube elongation and guidance defects
