# Supplementary material for: Spatiotemporal expression of regulatory kinases directs the transition from mitosis to cellular morphogenesis in Drosophila
Source: Nat Commun. 2022 Feb 9;13:772. doi: 10.1038/s41467-022-28322-8 (PMC8828718; doi:10.1038/s41467-022-28322-8)
Supplement: Supplementary file 3 — Description of additional supplementary files [file 41467_2022_28322_MOESM3_ESM.pdf]

Supplementary Data Titles

Supplementary Data 1.

Embryonic expression patterns of *Drosophila* and zebrafish protein kinases.

Supplementary Data 2.

Mass spectrometry results, related to Figure 3A.

Supplementary Data 3.

Source information for reagents.

Supplementary Movie Captions

Supplementary Movie 1. Bsd regulates myotube guidance.

Live imaging of LO1 myotubes from Stage 12 *slou>eGFP,nRFP* embryos. *bsd*^{1} myotubes failed to elongate anteriorly and attached at the posterior of the segment. GFP (green), RFP (violet). Arrowheads denote the myotube dorsal leading edge.

Supplementary Movie 2. Bsd does not regulate tendon cell number or position.

Live imaging of tendon cells from Stage 12 *sr>eGFP* embryos. Tendon cells in *bsd*^{1} embryos were indistinguishable from wild-type embryos.

Supplementary Movie 3. Tum regulates myotube guidance.

Live imaging of an LO1 myotubes from Stage 12 *tum*^{DH15} *slou>eGFP* embryos. *tum*^{DH15} myotubes often elongated toward the incorrect muscle attachment site.

Supplementary Movie 4. Microtubule reorganization is delayed in *bsd*^{1} myotubes. Live imaging of LO1 myotubes from Stage 12 *slou>Nod.GFP,LifeAct.RFP* embryos. Nod.GFP localizes to microtubule minus-ends; LifeAct localizes to F-actin. The formation of linear minus-end arrays was delayed in *bsd*^{1} myotubes. GFP (green), RFP (violet).
