# Supplementary material for: Spatiotemporal expression of regulatory kinases directs the transition from mitosis to cellular morphogenesis in Drosophila
Source: Nat Commun. 2022 Feb 9;13:772. doi: 10.1038/s41467-022-28322-8 (PMC8828718; doi:10.1038/s41467-022-28322-8)
Supplement: Supplementary file 11 — Reporting Summary [file 41467_2022_28322_MOESM11_ESM.pdf]

nature portfolio

Corresponding author(s): Aaron Johnson

Last updated by author(s): 2021/11/29

Reporting Summary

Nature Portfolio wishes to improve the reproducibility of the work that we publish. This form provides structure for consistency and transparency in reporting. For further information on Nature Portfolio policies, see our Editorial Policies and the Editorial Policy Checklist.

Statistics

For all statistical analyses, confirm that the following items are present in the figure legend, table legend, main text, or Methods section.

n/a	Confirmed	
☐	☒	The exact sample size (n) for each experimental group/condition, given as a discrete number and unit of measurement
☐	☒	A statement on whether measurements were taken from distinct samples or whether the same sample was measured repeatedly
☐	☒	The statistical test(s) used AND whether they are one- or two-sided<br>*Only common tests should be described solely by name; describe more complex techniques in the Methods section.*
☒	☐	A description of all covariates tested
☐	☒	A description of any assumptions or corrections, such as tests of normality and adjustment for multiple comparisons
☐	☒	A full description of the statistical parameters including central tendency (e.g. means) or other basic estimates (e.g. regression coefficient) AND variation (e.g. standard deviation) or associated estimates of uncertainty (e.g. confidence intervals)
☐	☒	For null hypothesis testing, the test statistic (e.g. F, t, r) with confidence intervals, effect sizes, degrees of freedom and P value noted<br>*Give P values as exact values whenever suitable.*
☒	☐	For Bayesian analysis, information on the choice of priors and Markov chain Monte Carlo settings
☒	☐	For hierarchical and complex designs, identification of the appropriate level for tests and full reporting of outcomes
☒	☐	Estimates of effect sizes (e.g. Cohen's d, Pearson's r), indicating how they were calculated

Our web collection on statistics for biologists contains articles on many of the points above.

Software and code

Policy information about availability of computer code

Data collection	Zen2; ZEISS<br>Applied Biosystems StepOne Real-Time PCR Systems software; Thermo Fisher
Data analysis	Adobe Creative,Cloud; Adobe<br>ClustalX; UCD<br>Origin 2019; Origin Lab<br>Office 365; Microsoft<br>Prism version 8; GraphPad Software Inc<br>DNAMAN; Lynnon Corporation

For manuscripts utilizing custom algorithms or software that are central to the research but not yet described in published literature, software must be made available to editors and reviewers. We strongly encourage code deposition in a community repository (e.g. GitHub). See the Nature Portfolio guidelines for submitting code & software for further information.

Data

Policy information about availability of data

All manuscripts must include a data availability statement. This statement should provide the following information, where applicable:

- Accession codes, unique identifiers, or web links for publicly available datasets
- A description of any restrictions on data availability
- For clinical datasets or third party data, please ensure that the statement adheres to our policy

The data that support all experimental findings of this study are available. Raw data necessary to reproduce all statistical analyses and results in the paper are provided in the Source Data File provided with this paper. Raw data is available in a publicly accessible repository.

Field-specific reporting

Please select the one below that is the best fit for your research. If you are not sure, read the appropriate sections before making your selection.

[x] Life sciences [ ] Behavioural & social sciences [ ] Ecological, evolutionary & environmental sciences

For a reference copy of the document with all sections, see nature.com/documents/nr-reporting-summary-flat.pdf

Life sciences study design

All studies must disclose on these points even when the disclosure is negative.

Sample size	Sample sizes were based on our previous, published sample sizes (PMCID 26293307).
Data exclusions	No data were excluded from our analyses.
Replication	In vitro assays were performed in triplicate and the mean +/- standard deviation is reported for each assay. All attempts at replication were successful.
Randomization	Samples were allocated into experimental groups based on genotype or treatment.
Blinding	Investigators were blinded to group allocation during data analysis.

Reporting for specific materials, systems and methods

We require information from authors about some types of materials, experimental systems and methods used in many studies. Here, indicate whether each material, system or method listed is relevant to your study. If you are not sure if a list item applies to your research, read the appropriate section before selecting a response.

Materials & experimental systems

n/a	Involved in the study	
[ ]	[x]	Antibodies
[ ]	[x]	Eukaryotic cell lines
[x]	[ ]	Palaeontology and archaeology
[ ]	[x]	Animals and other organisms
[x]	[ ]	Human research participants
[x]	[ ]	Clinical data
[x]	[ ]	Dual use research of concern

Methods

n/a	Involved in the study	
[x]	[ ]	ChIP-seq
[x]	[ ]	Flow cytometry
[x]	[ ]	MRI-based neuroimaging

Antibodies

Antibodies used

Anti-Bsd,This paper,NA,IHC (1:250); WB (1:500)
Anti-Mef2,R. Cripps,NA,IHC (1:1000)
Anti-GFP,Torrey Pines Biolabs,Cat#: TP401,IHC (1:600)
Anti-GFP,Aves Labs,Cat#: GFP-1020,IHC (1:300)
Anti-dsRED,Takara,Cat#: 632392,IHC (1:300)
Anti-beta-gal,Promega,Cat#: 926–32210,IHC (1:100)
Anti-FLAG,Sigma,Cat#: F3165,WB (1:1000)
Anti-Myc,Sigma,Cat#: PLA001,WB (1:1000)
Anti-gamma-tubulin,Sigma,Cat#: T5326,IHC (1:300)
Anti-alpha-Actinin,Sigma,Cat#: A7811,WB (1:1000)

Anti-Dig,Roche,Cat#: 11207733910,IHC (1:500)
Anti-GST,Cell signaling technology,Cat#: 2625,WB (1:1000)
Anti-Phosphothreonine,Abcam,Cat#: ab9337,WB (1:125)
Anti-Tropomyosin,Abcam,Cat#: MAC141,IHC (1:600)
Anti-PLK1-phospho-T210,Abcam,Cat#: ab39068,IHC (1:100); WB (1:500)
Goat anti-Chicken IgY, Secondary Antibody, HRP,Thermo Fisher,Ca#: PA1-28798,IHC (1:200)
Horse anti-Mouse IgG, Secondary Antibody, HRP,Cell signaling technology,Ca#: 7074,IHC (1:100); WB (1:1000)
Goat anti-Rabbit IgG, Secondary Antibody, HRP,Cell signaling technology,Ca#: 7074,IHC (1:100); WB (1:1000)
Goat anti-Mouse IgG, Secondary Antibody, Alexa Flour 488-conjugated ,Jackson ImmunoResearch INC,Ca#: 133733,IHC (1:50)
Goat anti-Rabbit IgG, Secondary Antibody, Alexa Flour 488-conjugated,Jackson ImmunoResearch INC,Ca#: 135742,IHC (1:50)
Goat anti-Rat IgG, Secondary Antibody, Alexa Flour 594-conjugated,Jackson ImmunoResearch INC,Ca#: 154060,IHC (1:50)
Goat anti-Mouse IgG, Secondary Antibody, Alexa Flour 594-conjugated,Jackson ImmunoResearch INC,Ca#: 133548,IHC (1:50)

Validation

Validation of the primary antibody we developed for this project (anti-Bsd) is shown in Supplemental Figure 2. The anti-gamma-tubulin antibody was previously reported (PMCID 2674253). The anti-Tropomyosin antibody was previously reported (PMCID 26293307). The anti-PLK1-phospho-T210 antibody was previously reported (PMCID 22291575).

Eukaryotic cell lines

Policy information about cell lines

Cell line source(s)

C2C12 and HEK293 cells were acquired from ATCC. S2 cells were from Paul H. Taghert.

Authentication

C2C12 cells were authenticated by differentiating cells into myotubes. The remaining cell lines were authenticated visually.

Mycoplasma contamination

Cell lines were not tested for mycoplasma contamination.

Commonly misidentified lines
(See ICLAC register)

none

Animals and other organisms

Policy information about studies involving animals; ARRIVE guidelines recommended for reporting animal research

Laboratory animals

Drosophila melanogaster was the only species of animal studied. All strains and sources are reported in Table S3. Male and female embryos were randomly used in this study.
aurA[1] BDSC "RRID: BDSC_3319 "
jar[322] BDSC "RRID: BDSC_8776 "
lost[1] BDSC "RRID: BDSC_57695 "
polo[1] BDSC "RRID: BDSC_546 "
polo[KG03033] BDSC "RRID: BDSC_13941 "
tum[DH15] BDSC "RRID: BDSC_8687 "
pav[B200] BDSC RRID: BDSC_4384
Df(2R)BSC199 BDSC "RRID: BDSC_9626 "
Df(2R)BSC699 BDSC "RRID: BDSC_26551 "
Df(3L)BSC447 BDSC "RRID: BDSC_24951 "
Df(2L)Exel7049 BDSC "RRID: BDSC_7821 "
P{UAS-nod.GFP} BDSC "RRID: BDSC_9282 "
P{Gal4-tey5053A} BDSC "RRID: BDSC_2702 "
P{GMR40D04-GAL4}attP2 (slou.Gal4) BDSC "RRID: BDSC_91545 "
P{Gal4-how24B} BDSC "RRID: BDSC_1767 "
P{UASp-aurB.PrA} BDSC "RRID: BDSC_85301 "
P{UAS-eGFP} BDSC "RRID: BDSC_5431 "
P{UAS-Lifeact-RFP} BDSC "RRID: BDSC_58362 "
P{UAS-polo.T182D} BDSC "RRID: BDSC_8434 "
P{PTT-GC}polo[CC01326] BDSC "RRID: BDSC_51552 "
P{UAS-GFP.nE2f1.1-230 } BDSC "RRID: BDSC_55100 "
P{UAS-RFP.CycB.1-266} BDSC "RRID: BDSC_55100 "
aurB[1689] Jean-René HUYNH
P{Gal4-kirrerP298} M Takeichi
P{kirrerP298.nlacZ} M Takeichi
P{Gal4-Mef2} E.N. Olson
P{MHC.tGFP} E.N. Olson

Wild animals

The study did not involve wild animals.

Field-collected samples

The study did not involve samples from the field.

Ethics oversight

Ethical guidance is not required for studies of invertebrate animals.

Note that full information on the approval of the study protocol must also be provided in the manuscript.
